# The Surgical Odyssey: Romania’s Contributions to Pituitary Gland Procedures

**DOI:** 10.3390/brainsci13101431

**Published:** 2023-10-08

**Authors:** Corneliu Toader, Andrei-Adrian Popa, Razvan-Adrian Covache-Busuioc, Bogdan-Gabriel Bratu, Alexandru Vlad Ciurea

**Affiliations:** 1 Department of Neurosurgery, National Institute of Neurology and Neurovascular Diseases, 077160 Bucharest, Romania; 2 Department of Neurosurgery “Carol Davila”, University of Medicine and Pharmacy, 020021 Bucharest, Romania; razvan-adrian.covache-busuioc0720@stud.umfcd.ro (R.-A.C.-B.); bogdan.bratu@stud.umfcd.ro (B.-G.B.); prof.avciurea@gmail.com (A.V.C.); 3 Department of Neurosurgery, Sanador Clinical Hospital, 010991 Bucharest, Romania; 4 Medical Science Section, Romanian Academy, 060021 Bucharest, Romania

**Keywords:** pituitary gland, Romanian physicians, surgical approach, hypophysectomy, Nicolae Paulescu, Gheorghe Marinescu, Grigore T. Popa, medical history, in vivo, hemorrhage, subtemporal approach, oral approach, cranial approach, sphenopalatine fossa

## Abstract

The pituitary gland, a puzzling medical subject up until the 20th century, had its early pathologies first documented in the 19th century by Pierre Marie and Hutchinson, where the gland’s meaningful study was hindered by its hard-to-reach location. This paper revisits the pioneering work of Romanian doctors such as Gheorghe Marinescu, Nicolae Paulescu, and Grigore T. Popa in surgical techniques targeting the pituitary gland. Marinescu’s 1892 experiment, albeit unsuccessful, laid the groundwork for future research in this area. Before Paulescu, surgical attempts could be classified into three types: oral, cranial, and sphenopalatine fossa approaches—all of which were notably dangerous and often resulted in fatal bleeding. Paulescu was the first to successfully and safely perform a complete in vivo hypophysectomy, opting for an innovative subtemporal method. He also conducted extensive research over four years to identify the gland’s essential functions. Later, a 1938 study by Popa and Harris demonstrated a temporal approach to the hypothalamo-hypophysial region in a rabbit. These groundbreaking contributions significantly influenced the trajectory of pituitary gland surgery.

## 1. Early Milestones in Pituitary Gland History

**Figure 1:**
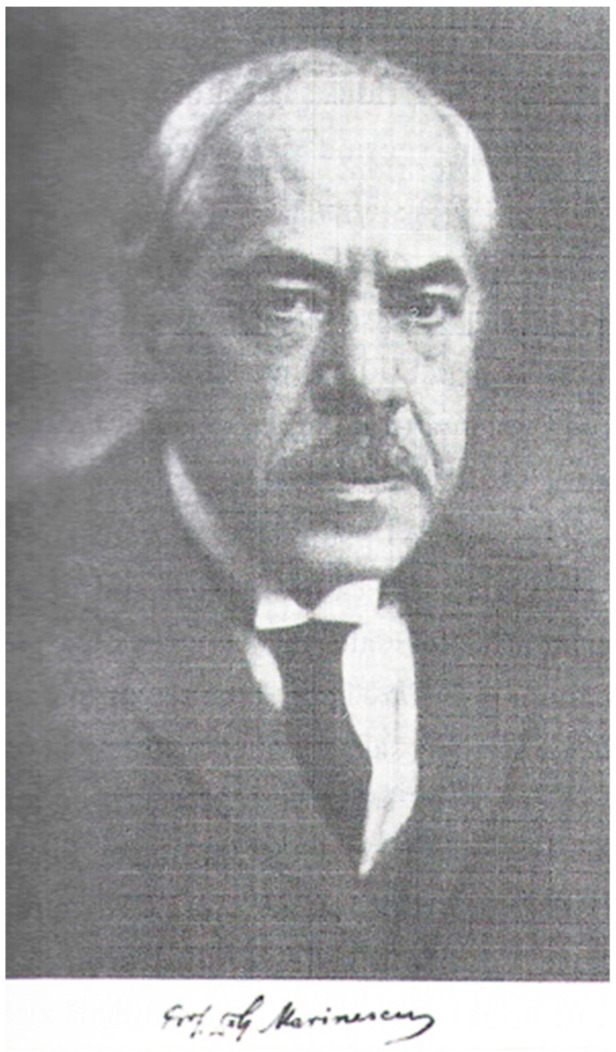
Gheorghe Marinescu (1863–1938).

The initial investigations into the role of the pituitary gland in human physiology were predominantly symptomatic in nature, focusing on the clinical manifestations resulting from the gland’s dysfunction. In the late 19th century, a pioneering revelation was made by French physician Pierre Marie, who identified acromegaly as a distinct pathological condition [1]. While Marie initially failed to discern the explicit relationship between acromegaly and pituitary abnormalities, it was in the year 1890 that a seminal publication was released, co-authored by Marie and Romanian neurologist Gheorghe Marinescu (1863–1938) (Figure 1).

This body of work was instrumental in delineating the anatomopathological features of acromegaly, specifically emphasizing its correlation with perturbations in pituitary morphology, including glandular hyperplasia and interstitial sclerosis. Although Marie and Marinescu initially postulated that acromegaly was the result of pituitary insufficiency rather than hyperfunction, the crux of their contributions lay in establishing the fundamental link between the pituitary gland and acromegaly, thereby catalyzing subsequent research trajectories within endocrinology and neurobiology [1,2].

The scholarly pursuits of Gheorghe Marinescu did not culminate with this collaborative endeavor; he continued to expand upon his initial findings. In 1892, Marinescu published another pivotal paper detailing an experimental hypophysectomy performed on a feline subject. Utilizing a transpalatal approach, he employed thermal cauterization to excise the pituitary gland. While the experiment was compromised by subsequent infection, Marinescu concluded that his data corroborated the feasibility of pituitary gland ablation through an oral route in felines, sustaining survival for a period of several weeks post-operation. The methodological shortcomings notwithstanding, Marinescu’s experiment provided invaluable impetus for future investigations within the realm of pituitary research.

Later citations of Marinescu’s work, particularly by researchers such as Paulescu, underscore its impact on the field. Paulescu recognized Marinescu as the inaugural investigator to physically interact with, and experimentally manipulate, the pituitary gland. He further noted that Marinescu’s experimental endeavors lent credence to the hypothesis—initially proposed in conjunction with Pierre Marie—that pituitary gland alterations could potentially be implicated in the pathogenesis of acromegaly [3]. Thus, the collaborative and independent contributions of Pierre Marie and Gheorghe Marinescu served as cornerstones in the foundational understanding of pituitary gland function and its role in systemic endocrine disorders, notably acromegaly.

The scientific inquiry into the pituitary gland presented substantial methodological challenges, owing in part to the anatomical complexity and inaccessibility of its location within the cranial cavity. Early attempts to approach this endocrine organ surgically were fraught with difficulty, primarily because the surgical techniques available at the time risked damaging adjacent neurological structures. One of the earliest recorded endeavors to surgically access a pituitary adenoma was undertaken by Sir Victor Horsley in 1889. Despite his pioneering efforts, the procedure was ultimately deemed unsuccessful due to excessive force applied during the retraction of the frontal and temporal lobes, which led to undesirable collateral damage [4].

**Figure 2:**
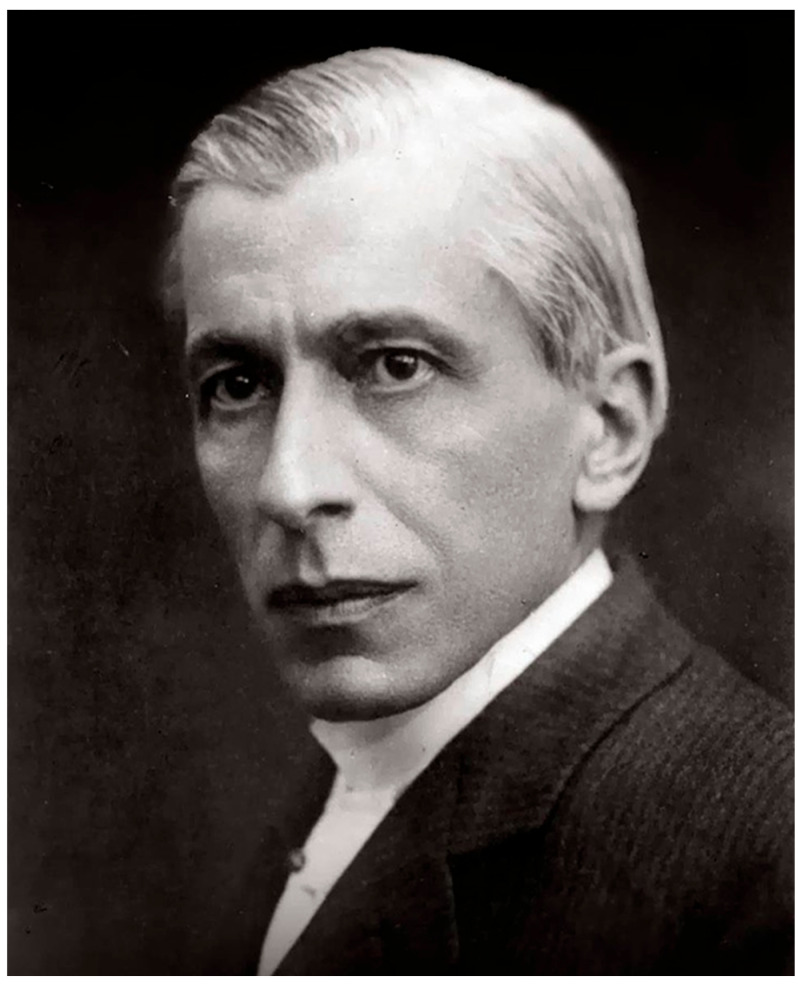
Nicolae Paulescu (1869–1931).

In 1903, a momentous leap in pituitary research was achieved by Romanian physiologist Nicolae Paulescu (1869–1931) (Figure 2). By employing a methodological approach that was meticulous enough to avoid the complications that had plagued earlier attempts, Paulescu successfully performed a total pituitary ablation on a canine subject. Subsequently, in his 1907 publication, he conclusively demonstrated the irreplaceable role of the pituitary gland in sustaining life [5].

The progression of surgical methodologies seeking to access the pituitary gland continued to evolve during the early 20th century. In 1907, Hermann Schloffer (1868–1937) introduced the first trans-sphenoidal approach to address pituitary growths, thereby offering an alternative that mitigated some of the risks associated with intracranial procedures [6]. This method was further refined by Harvey Cushing (1869–1939), an American neurosurgeon who became a seminal figure in pituitary research. Cushing initially perfected the trans-sphenoidal approach through a sublabial route, chronicling his findings in a series of publications released in 1912, 1914, and 1922. However, in 1929, he transitioned to a different surgical approach, the transfrontal method, adopting this subfrontal route as his exclusive technique for pituitary adenomectomy [7].

The history of pituitary gland research represents a chronology of methodological refinement, evolving from initial efforts to understand its physiopathological role, through to the implementation of surgical interventions, ranging from subtemporal approaches to trans-sphenoidal partial lesionectomies, and finally, to subfrontal adenomectomies. This trajectory underscores the incremental advancements that have been made in both the conceptual understanding of the pituitary gland and the surgical techniques employed to study and treat it.

## 2. Pituitary Gland Surgery: The Enigma and the Skill

The genesis of Nicolae Paulescu’s (1869–1931) scholarly interest in the pituitary gland remains an unchronicled facet of his career. Nonetheless, this curiosity led him to devise an idiosyncratic surgical method to access this endocrine organ: the transtemporal approach. Paulescu’s journey in developing effective surgical approaches for pituitary gland research can be seen as a series of methodological explorations, each with its own distinct advantages and shortcomings.

Initially, Paulescu, in collaboration with Dr. Paul Reyener, experimented with an oral approach to studying the pituitary gland. Conducted in Paris during the years 1897 and 1898, within the physiology laboratories of the Sorbonne, these experiments utilized canine subjects. Regrettably, the outcomes were suboptimal, with all animal subjects succumbing to either hemorrhage or suppurative meningeal infection. Given the catastrophic results, the investigators ultimately decided to discontinue this line of surgical approach.

In addition to the oral approach, other surgical routes were also carefully scrutinized and ultimately deemed untenable for varying reasons. For instance, a cranial approach via the upper part of the skull was dismissed by Paulescu, due to its propensity to induce extensive cerebral lesions. The sphenopalatine approach, although contemplated, was similarly ruled out, primarily because it did not afford an adequate visualization of the gland and carried an elevated risk of neurological damage.

Consequently, Paulescu opted for an innovative surgical route, the subtemporal approach, which emerged as a more viable option given the limitations of alternative techniques. After a series of preliminary attempts that commenced in Paris during the years 1897 and 1898, Paulescu successfully executed a complication-free total pituitary ablation on a canine subject on 11 March 1903. The operation was facilitated by the surgical expertise of Dr. Balacescu.

Paulescu’s contributions to pituitary gland research can be viewed as a meticulously constructed iterative process. Beginning with an exploration of various surgical approaches, each with distinct challenges and limitations, he ultimately devised the subtemporal approach, which demonstrated both the feasibility and a reduced risk of complications. This methodological refinement represents a significant milestone in the annals of endocrinology and neurosurgery, setting the stage for further advancements in our understanding of pituitary physiology and pathology.

**Figure 3:**
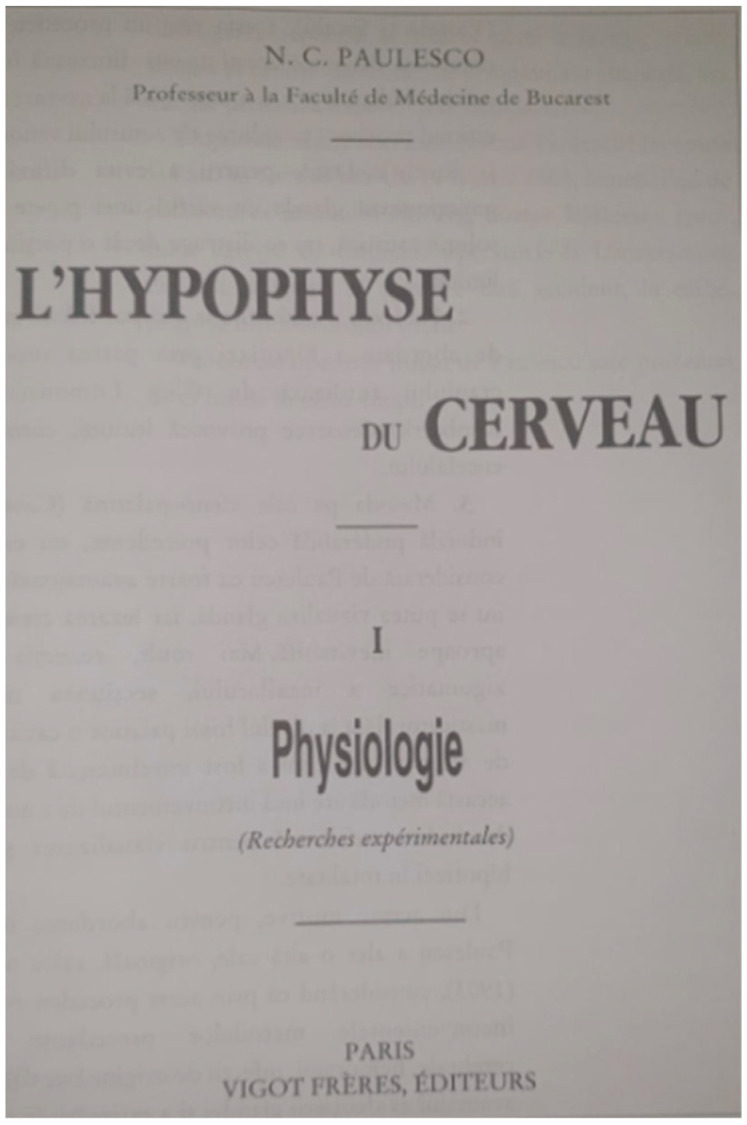
Paulescu’s work: *L’Hypophyse du Cerveau*—1908.

Nicolae Paulescu elucidated his pioneering surgical methodology for accessing the pituitary gland in a seminal work entitled *L’Hypophyse de Cerveau,* published in 1908 (Figure 3). The operative procedure was delineated in nine elaborate steps, providing a comprehensive framework for pituitary ablation [5].

The nine steps of Paulescu’s operative procedure for accessing the pituitary gland are as follows:

1. Cutaneous Incision and Muscle Dissection: The procedure begins with a midline incision on the skin, commencing slightly below the eyebrows and extending posteriorly to a few finger-widths behind the occipital protuberance. The platysma muscle, which adheres to the deep face of the skin, is longitudinally sectioned and disinserted. Sterilized compresses are employed to secure the skin edges.
2. Temporal Muscle Incision: On the left temporal region, a semicircular incision parallel to the superior insertion of the temporal muscle is made, extending from the external orbital apophysis to 2–3 cm below and lateral to the occipital protuberance. Subsequent dissection reveals the underlying temporal bone, and this is mirrored on the right side with slight variations.
3. Zygomatic Arch Manipulation: The zygomatic arch on the right side is cut at both ends and maneuvered outward and downward. This affords greater surgical accessibility to the temporal region.
4. Cranial Trepanation: A trephine is employed to create holes in the parietal bones on either side, through which further bone sections are made. Specialized forceps are used to minimize diploic hemorrhage.
5. Dura Mater Incision: A longitudinal incision is made on the dura mater, parallel to the previous bone incisions, creating a dural flap.
6. Temporal Lobe Elevation: A specialized instrument is inserted beneath the right temporal lobe to elevate it gently. The pituitary gland, recognizable by its characteristic red-yellow hue, becomes visible once the lobe is sufficiently elevated.
7. Pituitary Gland Extraction: Employing a specialized curette, the pituitary gland is carefully detached from its posterior anchoring and then fully excised from the base of the skull.
8. Dural and Muscular Closure: After pituitary removal and hemostasis, the dural flap is repositioned (Paulescu initially used thin catgut sutures for this, but later abandoned them, deeming them unnecessary). The zygomatic arch and temporal muscle are then sutured back into place.
9. Final Closure and Dressing: The incisions are sutured and sterilizing compresses are applied to the surgical site. Special attention is given to protect the eyes and ears of the animal with sterilizing cotton wool, and a compressive dressing is applied to the head, allowing unimpeded respiration and deglutition.

This elaborate surgical schema, developed by Paulescu, marked a significant advancement in the field of neuroendocrinology, providing a sophisticated and structured approach for pituitary gland access and removal. The methodological rigor of Paulescu’s operative procedure laid the groundwork for subsequent innovations in pituitary surgery and significantly enhanced our understanding of endocrine physiology and pathology.

## 3. Pioneering Anesthesia in Pituitary Gland Surgery

In the early 20th century, Nicolae Paulescu developed an innovative anesthesia protocol to facilitate his groundbreaking pituitary surgeries. He initially induced anesthesia using ether and then maintained the narcotic state with periodic administrations of chloroform. Paulescu found that this dual-agent approach mitigated complications and appeared to reduce the tendency for hemorrhages as compared to using ether alone. This practice was thoroughly documented in 1908, subsequent to its initial employment in 1907 [5].

Paulescu’s surgical technique was via a transtemporal approach, wherein the temporal lobe was carefully moved to provide access to the pituitary gland. His seminal research yielded compelling evidence for the critical role of the pituitary in maintaining life. Paulescu found that all dogs subjected to total pituitary ablation died within 24 hours post-surgery. However, some animals survived considerably longer after partial hypophysectomy—two lived for five months, and another for a year. These observations led him to conclude that the pituitary gland is indeed indispensable for life.

In the following years, Harvey Cushing and other leading neurosurgeons, including Crowe and Homans in 1910 and Reford in 1909, replicated Paulescu’s pioneering work. They utilized Paulescu’s surgical techniques to perform an extensive series of hypophysectomies on dogs [8]. The results corroborated Paulescu’s conclusions: the pituitary gland was crucial for survival.

The validation of Paulescu’s work by Cushing and his team not only lent credibility to Paulescu’s groundbreaking contributions, but also amplified the global understanding of the critical role of the pituitary gland in physiological regulation. Collectively, these early surgical explorations laid an important foundation for the modern field of neuroendocrinology, demonstrating the pituitary’s indispensable role in sustaining life and paving the way for subsequent advancements in both surgical techniques and endocrine therapies.

## 4. The Legacy of a Lost Approach: A Timely Reassessment

Although Nicolae Paulescu’s subtemporal approach to the pituitary gland marked a milestone as the first in vivo approach with long-term survival and without complications, it did not become the primary surgical technique for addressing pituitary pathology. Instead, Harvey Cushing and Oskar Hirsch pioneered the trans-sphenoidal approach in 1909, which later gained wider acceptance [7].

Cushing acknowledged Paulescu’s contributions in his own reports, particularly emphasizing Paulescu’s discovery that retaining a fragment of the pituitary gland is essential for survival [8]. The significance of Paulescu’s work was also recognized by Sir Sharpey Shafer in his 1926 treatise, “The Endocrine Organs”. According to Shafer, Paulescu was the first to definitively show that the complete removal of the pituitary gland results in fatal outcomes. This landmark finding has been verified across various classes of vertebrates and was subsequently confirmed by Cushing and other researchers, including Bield, B.A. Houssay, Asoli and Lagnani, Blair Bell, and Dotti.

Further cementing Paulescu’s legacy, Evelyn Anderson and Webb Haymaker highlighted his contributions in their 1974 treatise, “Progress in Brain Research,” particularly in the chapter on “Hypothalamic and Pituitary Research”. According to them, Paulescu was among the early trailblazers in the field and was notably the first to develop a superior operative technique for pituitaryectomy in dogs [9].

Overall, although Paulescu’s subtemporal approach may not have been adopted as the primary surgical technique for treating pituitary disorders, his groundbreaking work established critical principles concerning the gland’s physiological importance and introduced a pioneering surgical method. His contributions have been acknowledged by subsequent generations of researchers and clinicians, affirming his role in the historical development of neuroendocrinology and pituitary surgery.

## 5. Temporal Approach—Revisiting Traditional Views

Another significant step forward in the evolution of experimental research with regard to the pituitary gland took place in 1938, with the work of Grigore T. Popa and Geoffrey Wingfield Harris. Popa had previously gained attention for his landmark publication in *The Lancet* in 1930, which was co-authored with Una Fielding, where he described the hypothalamo-hypophyseal portal system for the first time. This discovery set the stage for his later work with Harris, aiming to improve access to the hypothalamo-hypophyseal region for further study [10].

Moreover, their 1938 publication, *A Technique for Operations on the Hypothalamo-Hypophysial Region of the Rabbit* [10], introduced a four-stage temporal approach on rabbits to better expose the region of interest:

First Stage: The authors initiated the process with the surgical removal of a part of the zygomatic arch. The skin and superficial fascia were incised, and the periosteum over the zygomatic arch was carefully reflected. The arch was then removed, with caution taken not to damage large vessels in the masseter muscle. If hemorrhage occurred, it could be controlled by applying pressure.
Second Stage: The procedure continued with the removal of the upper ramus of the mandible. The overlying masseter muscle was cut, and the periosteum was removed. The mandibular articular surface was separated from the mandible through gentle pressure, followed by a bone cut.
Third Stage: The next stage involved exposing and removing part of the squamous temporal bone. The pterygomaxillary fossa was cleared, and the skull was accessed in the temporo-sphenoidal region. Once reached, the dura mater was incised and reflected to expose the underlying structures.
Fourth Stage: The final stage involved retracting the temporal lobe of the brain to expose the hypothalamo-hypophyseal region. Upon retraction, the tentorium cerebelli became visible, and the hypothalamus could be identified above it. In this exposed region, the small internal carotid artery was visible, and the hypophyseal stalk was located postero-medially to this artery.

The research by Popa and Harris further advanced the field’s understanding of the hypothalamo-hypophyseal system and introduced a new surgical technique that facilitated the exploration of this critical region. Their work stands as a notable contribution to the lineage of research that aims to better understand the physiological and anatomical complexities of the pituitary gland and its surrounding structures.

## 6. Conclusions

Initially regarded as a relatively unimportant anatomical structure, the pituitary gland’s significance has become increasingly evident, thanks to the seminal contributions of pioneering researchers and surgeons. The evolving understanding of this essential gland is a story of intellectual pursuit, marked by breakthroughs and advances that have fundamentally shifted our understanding of its role in physiology.

The journey to unravel the complexities of the pituitary—also known as the hypophysis—began with early trailblazers like Pierre Marie and Gheorghe Marinescu. They laid the initial groundwork by investigating the correlation between acromegaly, a disorder characterized by the abnormal growth of bones and tissues, and the pituitary gland. Their work hinted at the critical functions that this once-overlooked organ might serve, setting the stage for further investigations.

Subsequently, Nicolae Constantin Paulescu emerged as a key figure who dispelled the notion of the pituitary as an inconsequential organ. Through rigorous experimentation, Paulescu conclusively demonstrated that the pituitary is indispensable for life itself. His surgical techniques for pituitaryectomy in dogs paved the way for more in-depth studies, effectively shifting the perception of the gland from that of a mere anatomical curiosity to an organ of vital importance.

The story, however, does not end with Paulescu. Surgeons and researchers like Victor Horsley and Hermann Schloffer carried the baton forward, each contributing valuable insights and methodologies. However, it was perhaps Harvey Cushing who most dramatically advanced the field by pioneering new surgical approaches to pituitary disorders. His work, in collaboration with others on his team, not only validated earlier findings pertaining to the pituitary’s essential functions, but also opened a new chapter in medical history—the era of surgical interventions focused on the pituitary gland.

Thus, what began as an exploration of a seemingly inconsequential organ eventually evolved into a rich, multidisciplinary field of study. Today, the pituitary is recognized not merely as a gland, but as a cornerstone of the endocrine system, influencing a range of physiological processes, from growth and metabolism to the functioning of other glands. This sea of change in pituitary gland knowledge is a testament to the generations of scholars and practitioners who dedicated themselves to unraveling the mysteries of the pituitary gland. Their collective work has not only deepened our biological understanding, but also introduced surgical and therapeutic options that continue to improve and save lives to this day.
